# Evidence of Submicroscopic Malaria Parasitemia, Soil-Transmitted Helminths, and Their Coinfections Among Forest-Fringed Orang Asli Communities in Peninsular Malaysia

**DOI:** 10.4269/ajtmh.24-0718

**Published:** 2025-04-08

**Authors:** Nurmanisha Abdull-Majid, Nan Jiun Yap, Mian Zi Tee, Yi Xian Er, Romano Ngui, Yvonne Ai-Lian Lim

**Affiliations:** ^1^ Department of Parasitology, Faculty of Medicine, Universiti Malaya, Kuala Lumpur, Malaysia;; ^2^ Department of Para-Clinical Sciences, Faculty of Medicine and Health Sciences, Universiti Malaysia Sarawak (UNIMAS), Sarawak, Malaysia

## Abstract

Malaysia’s malaria rate has declined but remains a public health concern, with limited investigations into malaria and coinfections with soil-transmitted helminth (STH) infections. A cross-sectional study using convenience sampling in Orang Asli villages enrolled 437 villagers aged 1–83 years based on their willingness to participate. Blood samples were tested microscopically for malaria, followed by nested polymerase chain reaction (PCR), and stool samples were screened microscopically for STH eggs. Body temperature, demographic, and socioeconomic data were collected. Malaria parasite was detectable only via PCR, with a 15.3% prevalence, indicating submicroscopic malaria parasitemia; none of the positive cases presented fever. The identified species included *Plasmodium vivax* (8.7%), *Plasmodium cynomolgi* (5.5%), *Plasmodium knowlesi* (4.3%), *Plasmodium falciparum* (1.8%), *Plasmodium inui* (0.2%), and *Plasmodium malariae* (0.2%). Females had significantly higher rates of submicroscopic malaria parasitemia (19.6%) compared with males (9.3%, *P* = 0.003). STH infections were highly prevalent (71.4%), with *Trichuris trichiura* (65.2%), *Ascaris lumbricoides* (35.0%), and hookworm (14.6%). STH infection was associated with age (*P* <0.001), peaking in individuals aged 10–19 years (86.2%) and 1–9 years (83.0%), as well as with students (84.3% versus 60.8% in employed and 60.3% in unemployed; *P* <0.001) and low-income households (76.4% versus 61.7% in higher-income households; *P* = 0.002). Submicroscopic malaria parasitemia and STH coinfections were present in 8.9% of participants, with higher rates in low-income households (12.6% versus 5.2% in higher-income, *P* = 0.010). The Negrito tribe exhibited the highest prevalence of submicroscopic malaria parasitemia, STH, and coinfections (*P* <0.05). This study highlights the need for integrated malaria and STH control strategies, particularly for the Negrito tribe.

## INTRODUCTION

The indigenous communities in Peninsular Malaysia, known as the Orang Asli, are a minority group that accounts for approximately 0.8% of the overall population of 32.4 million in Malaysia.^1^ Despite Malaysia’s rapid economic development and modernization, the Orang Asli remain among Malaysia’s most socioeconomically disadvantaged populations, facing significant health challenges, particularly a high burden of neglected tropical diseases (NTDs). Among the most common NTDs reported among Orang Asli are malaria and soil-transmitted helminth (STH) infections.^2^

Malaria, a vector-borne parasitic disease caused by *Plasmodium* spp., is transmitted to humans through the bite of infected female *Anopheles* mosquitoes. There are four species of malaria that are widely known to cause disease in humans: *Plasmodium falciparum, Plasmodium vivax, Plasmodium ovale, and Plasmodium malariae*. Since the introduction of the National Strategic Plan for Malaria Elimination, cases caused by the four human *Plasmodium* species dropped from over 5,000 in 2010 to zero indigenous cases in 2018.^3^ However, in recent years, evidence of simian malaria parasites infecting humans has been reported in Malaysia, including *Plasmodium knowlesi, Plasmodium cynomolgi, Plasmodium inui,* and *Plasmodium coatneyi.*^3^^–^^7^ This trend is attributed to deforestation and land clearing for agriculture or urban development, which particularly impact the Orang Asli communities who depend on forests for their livelihoods.

Most malaria prevalence studies focus on hospitalized clinical samples, which often overlook submicroscopic malaria cases. According to WHO,^8^ submicroscopic malaria refers to low-density blood-stage malaria infections that are not detected by conventional microscopy. These infections can be identified through highly sensitive methods such as polymerase chain reaction (PCR). Reports in Peninsular Malaysia highlight the presence of submicroscopic malaria parasitemia, suggesting its potential role as a reservoir for ongoing malaria transmission.^9^^–^^12^ Of greater concern, submicroscopic malaria parasitemia was observed with up to three mixed-species infections.^9^^,^^13^ This area is often underreported and warrants further investigation.

On the other hand, STHs are highly prevalent among Orang Asli communities with prevalence rates exceeding 50%.^14^^–^^17^ The most common STH species include *Trichuris trichiura* (whipworms), *Ascaris lumbricoides* (roundworms), and hookworms (*Ancylostoma duodenale* and *Necator americanus*).^18^ STH prevalence varies by region and population in Malaysia, with higher rates in areas with limited clean water, sanitation, hygiene facilities, and lower socioeconomic and educational status.^15^^,^^19^^,^^20^

The co-endemicity of malaria and STH infections in many tropical and subtropical regions has sparked considerable interest in their potential interactions. However, the relationship remains inconclusive: some studies indicate protective association,^21^^,^^22^ whereas others suggest that STH increases malaria risk and severity.^23^ Some studies report no significant association between the two parasitic infections.^24^ Despite the significance of this association, data on malaria–STH coinfection from Malaysia remains scarce. To our knowledge, only one study in Malaysia has investigated STH coinfection among hospital-based malaria patients, finding a 48.9% coinfection rate.^25^ However, it did not include *Trichuris trichiura* infection, *Plasmodium knowlesi,* other zoonotic malaria, infection intensity/density, and was limited to hospital cases. Thus, our study aims to assess the prevalence of community-based malaria, STH, and their coinfection among the at-risk communities in Malaysia. This research will add value to instituting effective control and prevention strategies for both infections.

## MATERIALS AND METHODS

### Study design.

**Figure 1.**
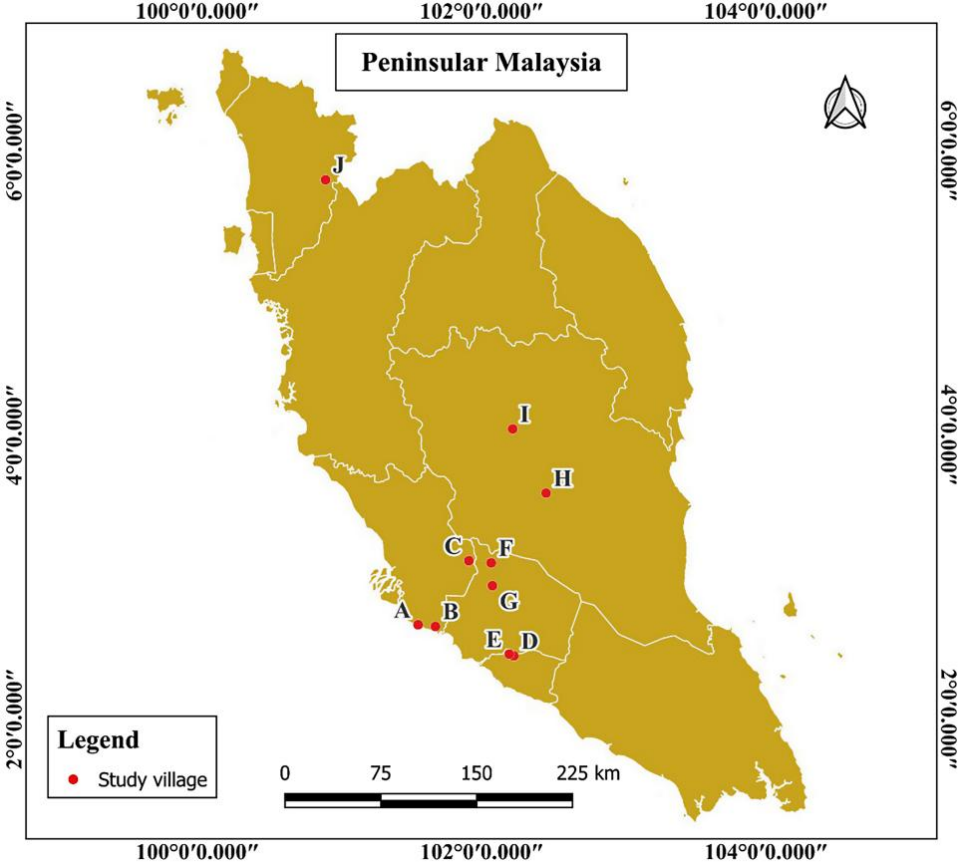
Map of Peninsular Malaysia showing the research study area.

This cross-sectional study was conducted among Orang Asli communities across Peninsular Malaysia, spanning 10 villages across the states of Negeri Sembilan, Melaka, Selangor, Pahang, and Kedah (Figure 1). Villages were selected based on convenience sampling from a list provided by the Department of Orang Asli Development (JAKOA), considering road accessibility and community willingness to participate, confirmed through prior discussions with village chieftains. These villages were located near forest edges and surrounded by agricultural estates, including rubber and oil palm plantations. Detailed information regarding the sample distribution across the study population is presented in Supplemental Table 1.

Participants were recruited from each village based on predefined inclusion and exclusion criteria. The inclusion criteria encompassed Orang Asli individuals across all age groups who provided paired blood and stool samples for analysis. Participants with a history of anthelmintic drug treatment in the previous 6 months were excluded, as this may affect baseline measurements.^26^

The study’s sample size was calculated using the formula described in Naing et al.,^27^ assuming a malaria–STH coinfection prevalence of 50% because of limited regional studies,^28^^,^^29^ with a 95% confidence level, 5% margin of error, and an additional 10% to account for possible nonresponse or loss. The minimum required sample size was 422.

### Sampling and data collection.

Community awareness sessions about the infections under study were organized in each village’s community hall, where researchers also explained the study in the local language. These sessions included demonstrations of proper stool collection techniques and explanations of the blood sampling procedure. All eligible villagers who were willing to participate were provided with pre-labeled stool containers containing their names and sample codes. For eligible villagers who were not present, at least two follow-up visits were made through house-to-house outreach at different times of the day. Upon receiving the stool samples, 6 mL of venous blood samples were drawn from each participant and collected into ethylenediaminetetraacetic acid (EDTA) tubes. Their body temperature, basic demographic information of the participants (age, gender, and ethnicity), and socioeconomic status (education level, employment status, and monthly household income) were also obtained and recorded.

### Laboratory procedure.

#### Microscopy screening of malaria.

Microscopic examinations, the gold standard for malaria diagnosis, were conducted according to WHO protocols.^30^ Thick and thin blood films were prepared from EDTA-collected blood samples at each study site. Thin films were fixed with absolute methanol, and both films were stained with 3% Giemsa for 30 minutes. Two independent personnel conducted microscopic examinations under an oil immersion at 100× magnification. A blood slide was deemed microscopy-negative if no *Plasmodium* parasites were observed in 100 microscope fields. Samples negative by microscopy were then tested using molecular detection methods, and any PCR-positive samples were classified as submicroscopic malaria parasitemia.

#### *Molecular detection of Plasmodium* spp. *using nested PCR.*

DNA extraction from whole blood samples was performed using the QIAamp DNA Blood Mini Kit (Qiagen, Germany), following the manufacturer’s protocol. Extracted DNA was then subjected to species-specific nested PCR targeting the *Plasmodium* spp. 18s ribosomal RNA gene. Initial screening for *Plasmodium* presence used genus-specific primers (rPLU1 and rPLU5 [nest 1]; rPLU3 and rPLU4 [nest 2]).^31^ Positive samples underwent a second round of PCR using species-specific primers to identify various *Plasmodium* species, covering both human malaria species (i.e., *Plasmodium falciparum*, *Plasmodium vivax*, *Plasmodium malariae*, and *Plasmodium ovale*)^31^ and simian malaria species (i.e., *Plasmodium knowlesi*,^32^ *Plasmodium coatneyi*,^33^ *Plasmodium cynomolgi*,^33^ *Plasmodium inui*,^33^ and *Plasmodium fieldi*).^33^ A full list of PCR primers sequences and protocols is provided in Supplemental Table 2. Each PCR run included negative and positive controls to validate the results. The PCR products were then visualized on a 2% agarose gel under electrophoresis condition at 90 volts for 45 minutes. Positive PCR products were sequenced bidirectionally to ensure precise identification of the *Plasmodium* species. The DNA sequence results were analyzed using basic local alignment search tool in the National Center for Biotechnology Information database.

#### Microscopy screening of STH infections.

All stool samples were processed using the formalin-ether sedimentation method, as described by Allen and Ridley,^34^ to detect the presence of STH species. This method demonstrated high sensitivity for detecting STH eggs, as reported in various studies.^35^^,^^36^ The modified Kato-Katz method, conducted in field settings, was also used to detect the presence and quantify the burden of STH infections.^37^ However, this method was only applied to 160 samples because of logistical constraints and limited resources in the field, which require immediate processing of fresh stool samples. Hookworm eggs were screened under a microscope after a clearing time of 30–60 minutes, as they tend to disintegrate early, whereas species were examined after 60 minutes. The prepared single slide was examined and cross-checked by another person. WHO classifies STH intensities by eggs per gram: *Ascaris lumbricoides* (light: 1–4,999, moderate: 5,000–49,999, heavy: >50,000), *Trichuris trichiura* (light: 1–999, moderate: 1,000–9,999, heavy: >10,000), and hookworm (light: 1–1,999, moderate: 2,000–3,999, heavy: >4,000).^38^ The result was considered positive when at least one STH egg was observed in either of these two techniques.

## STATISTICAL ANALYSES

Data completeness was verified before analysis, addressing any errors or missing information. The data were then analyzed using SPSS version 27.0 (IBM Corp., 2021, Armonk, NY). Cross-tabulation was applied to assess the frequency distributions of malaria occurrences. To test for associations between infections (malaria, STH, and coinfections) and demographic factors, χ^2^ or Fisher’s exact tests were performed as appropriate. A *P*-value of <0.05 was set as the threshold for statistical significance throughout the analyses.

## RESULTS

### Characteristics of the study population.

A total of 437 Orang Asli participants were enrolled in this study, with a median age of 18 years (ranging from 1 to 83 years old). The primary occupation was agricultural work (21.6%), whereas almost three-quarters were unemployed (71.9%), including housewives, students, and others not working. Household incomes below Ringgit Malaysia (RM) 800 were classified as lower income (50.8%), and those at or above RM 800 were classified as higher income (49.2%). The majority of participants had received at least a primary level of education (78.1%). Within this cohort, 53.3% belonged to the Proto-Malay tribe, 24.7% to the Negrito tribe, and 22.0% to the Senoi tribe. Table 1 provides further demographic details of the study participants.

**Table 1:** Demographic characteristics of study participants

Characteristics	% (*n*)
Gender	437
Male	41.6 (182)
Female	58.4 (255)
Age (years)	437
1–9	22.9 (100)
10–19	28.1 (123)
20–29	14.9 (65)
30–39	12.6 (55)
40–49	10.3 (45)
≥50	11.2 (49)
Occupation	426
Student	43.2 (184)
Agricultural worker	21.6 (92)
Housewife	19.5 (83)
Unemployed	9.2 (39)
Others (e.g., fisherman, bus driver, factory worker, government staff)	6.6 (28)
Monthly household income	392
≥RM 800	49.2 (193)
<RM 800	50.8 (199)
Education level	421
No formal education	21.9 (92)
Primary education	58.4 (246)
Secondary education	19.2 (81)
Tertiary education	0.5 (2)
Tribe	437
Proto-Malay	53.3 (233)
Negrito	24.7 (108)
Senoi	22.0 (96)

RM = Ringgit, Malaysia.

### Submicroscopic malaria parasitemia.

Despite thorough microscopic screening, no malaria parasites were visually identified on the collected slides, indicating an apparent absence of detectable parasites among the study participants. This finding was not unexpected, as none of the individuals exhibited signs of fever (≥37.5°C). However, nested PCR analysis, capable of detecting as few as six parasites per microliter of blood,^31^ confirmed the presence of submicroscopic malaria parasitemia in the samples. The prevalence of submicroscopic malaria parasitemia was 15.3% (67 of 437). Species-specific nested PCR successfully identified the species of *Plasmodium* in 53 samples, leaving 14 samples unidentifiable. Among the identified species, *Plasmodium vivax* was the most common (8.7%, 38 of 437), followed by *Plasmodium cynomolgi* (5.5%, 24 of 437), *Plasmodium knowlesi* (4.3%, 19 of 437), *Plasmodium falciparum* (1.8%, 8 of 437), *Plasmodium inui* (0.2%, 1 of 437), and *Plasmodium malariae* (0.2%, 1 of 437) (Table 2).

**Table 2:** Prevalence of submicroscopic malaria parasitemia among the studied population in Peninsular Malaysia

Type of Infections	% (*n*)	95% CI
Overall submicroscopic malaria parasitemia	67 (15.3)	12.1–19.1
Submicroscopic malaria parasitemia (by species)		
Pf	1.8 (8)	0.8–3.6
Pv	8.7 (38)	6.2–11.7
Pm	0.2 (1)	0.0–1.3
Pk	4.3 (19)	2.6–6.7
Pcy	5.5 (24)	3.6–8.1
Pi	0.2 (1)	0.0–1.3
Unidentified species	3.2 (14)	1.8–5.3
Single species submicroscopic malaria parasitemia		
Pf	0.7 (3)	0.1–2.0
Pv	3.7 (16)	2.1–5.9
Pm	0.2 (1)	0.0–1.3
Pk	0.7 (3)	0.1–2.0
Pcy	1.4 (6)	0.5–3.0
Pi	0.2 (1)	0.0–1.3
Mixed species submicroscopic malaria parasitemia		
Pf + Pv	0.2 (1)	0.0–1.3
Pv + Pk	0.9 (4)	0.2–2.3
Pv + Pcy	1.1 (5)	0.4–2.6
Pk + Pcy	0.2 (1)	0.0–1.3
Pf + Pv + Pcy	0.2 (1)	0.0–1.3
Pv + Pk + Pcy	1.8 (8)	0.8–3.6
Pf + Pv + Pk + Pcy	0.7 (3)	0.1–2.0

Pcy = *Plasmodium cynomolgi*; Pf = *Plasmodium falciparum*; Pi = *Plasmodium inui*; Pk = *Plasmodium knowlesi*; Pm = *Plasmodium malariae*; Pv = *Plasmodium vivax*.

In this study, mixed-species malaria infections (5.3%, 23 of 437) were less prevalent than single-species infections (6.9%, 30 of 437). Double infections were observed in 2.5% (11 of 437) of participants, followed by triple infections (2.0%, 9 of 437) and quadruple infections (0.7%, 3 of 437). The most common mixed infection was the combination of *Plasmodium vivax*, *Plasmodium knowlesi,* and *Plasmodium cynomolgi*, accounting for 1.8% (8 of 437). Mixed-species infections involving both human and zoonotic malaria were identified in 4.8% (21 of 437) of cases, a prevalence higher than that of mixed infections involving only human malaria species (0.2%, 1 of 437) or only zoonotic malaria species (0.2%, 1 of 437) (Table 2).

Table 3 shows that submicroscopic malaria parasitemia was significantly higher among females than males (*P* = 0.003). Among tribes, the Negrito tribe recorded significantly higher submicroscopic malaria parasitemia rates compared with the non-Negrito tribe (*P* = 0.001). Additionally, submicroscopic malaria parasitemia was recorded in participants across all age groups, with the highest prevalence observed among those aged 50 years and older.

**Table 3:** Prevalence of submicroscopic malaria parasitemia, STH and their coinfection according to demographic and socioeconomic factors

Variables	*N*	Submicroscopic Malaria Parasitemia		STH Infection		Coinfection	
		Positive, % (*n*)	*P*-Value	Positive, % (*n*)	*P*-Value	Positive, % (*n*)	*P*-Value
Demographic factors							
Gender	437						
Male	182	9.3 (17)	0.003*	74.2 (135)	0.277	6.6 (12)	0.149
Female	255	19.6 (50)		69.4 (177)		10.6 (27)	
Age group (years)	437						
1–9	100	10.0 (10)	0.129	83.0 (83)	<0.001*	8.0 (8)	0.639
10–19	123	12.2 (15)		86.2 (43)		8.9 (11)	
20–29	65	16.9 (11)		66.2 (43)		9.2 (6)	
30–39	55	18.2 (10)		54.5 (30)		5.5 (3)	
40–49	45	17.8 (8)		57.8 (26)		15.6 (7)	
≥50	49	26.5 (13)		49.0 (24)		8.2 (4)	
Tribe	437						
Negrito	108	25.0 (27)	0.001*	82.4 (89)	0.004*	21.3 (23)	<0.001*
Non-Negrito	329	12.2 (20)		67.8 (223)		4.9 (16)	
Socioeconomic factors							
Education	421						
No formal education	92	10.9 (10)	0.135	75.0 (69)	0.341	5.4 (5)	0.152
Receive formal education	329	17.3 (57)		69.9 (230)		10.3 (34)	
Employment status	426						
Used	120	15.8 (19)	0.079	60.8 (73)	<0.001*	10.8 (13)	0.658
Unemployed	121	21.5 (26)		60.3 (73)		7.4 (9)	
Students	185	11.9 (22)		84.3 (156)		9.2 (17)	
Monthly household income	392						
≥RM 800	193	13.0 (25)	0.126	61.7 (119)	0.002*	5.2 (10)	0.010*
<RM 800	199	18.6 (37)		76.4 (152)		12.6 (25)	

RM = Ringgit, Malaysia; STH = soil-transmitted helminth.

* Bold indicates significant association *P* <0.05.

### STH infections.

The prevalence of STH infections was 71.4% (312 of 437), with *Trichuris trichiura* being the most prevalent species (65.2%, 285 of 437), followed by *Ascaris lumbricoides* (35.0%, 153 of 437) and hookworm (14.6%, 64 of 437) (Table 4). The rate of mixed STH species infections (36.8%, 161 of 437) was slightly higher than that of single STH species infections (34.6%, 151 of 437). Among those with mixed species infections, 30.2% (132 of 437) had double infections, whereas 6.6% had triple infections. The most common mixed-species infection was the combination of *Ascaris lumbricoides* and *Trichuris trichiura*.

**Table 4:** Prevalence of STH infections among the studied population in Peninsular Malaysia

STH Species	% (*n*)	95% CI
Overall STH infection	71.4 (312)	66.9–75.6
STH infection (by species)		
*Ascaris lumbricoides*	35.0 (153)	30.5–39.7
Hookworm	14.6 (64)	11.5–18.3
*Trichuris trichiura*	65.2 (285)	60.5–69.7
STH single/mixed species		
*Ascaris lumbricoides*	4.8 (21)	3.0–7.3
Hookworm	0.5 (2)	0.1–1.6
*Trichuris trichiura*	29.3 (128)	25.1–33.8
*Ascaris lumbricoides* + hookworm	0.9 (4)	0.2–2.3
*Ascaris lumbricoides + Trichuris trichiura*	22.7 (99)	18.8–26.9
Hookworm + *Trichuris trichiura*	6.6 (29)	4.5–9.4
*Ascaris lumbricoides* + hookworm + *Trichuris trichiura*	6.6 (29)	4.5–9.4

STH = soil-transmitted helminth.

**Figure 2.**
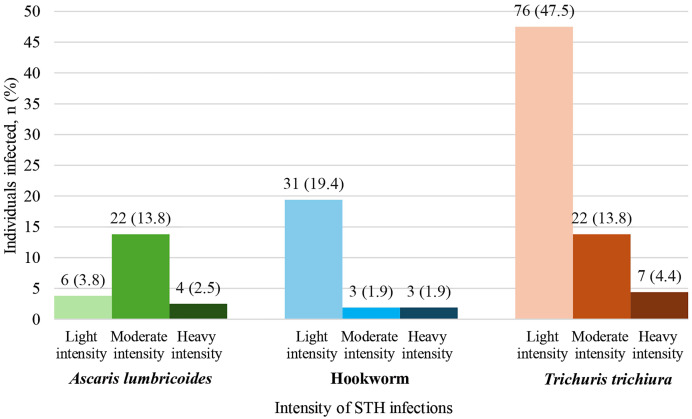
Intensity of soil-transmitted helminth (STH) infections (eggs per gram) among infected study participants.

Of the 160 samples with Kato-Katz intensity data, 106 (68.1%) were positive for STH. The prevalence of *Trichuris trichiura*, hookworm, and *Ascaris lumbricoides* was 65.6% (105), 23.1% (37), and 20.0% (32), respectively. Light infections of *Trichuris trichiura* were the most common (47.5%), followed by light infections of hookworm (19.4%) and moderate infections of *Ascaris lumbricoides* (13.8%) (Figure 2).

Table 3 shows a significant association between STH infection and age groups (*P* <0.001), with the highest prevalence in participants aged 10–19 years (86.2%, 43 of 123), closely followed by those aged 1–9 years (83.0%, 83 of 100). Additionally, students were associated with a higher prevalence of infections (*P* <0.001), and households with a monthly income below RM 800 had significantly higher STH infection rates than those with an income of RM 800 and above (*P* = 0.002). In terms of tribes, the Negrito tribe recorded significantly higher infection rates compared with the non-Negrito tribe (*P* = 0.004).

### Submicroscopic malaria parasitemia and STH coinfections.

Of the 437 study participants, 8.9% (39) were coinfected with both submicroscopic malaria parasitemia and STH, with the most common combination being malaria and *Trichuris trichiura* (7.8%, 34), followed by malaria and *Ascaris lumbricoides* (3.2%, 14) and malaria and hookworm (1.6%, 7). Each coinfection contained at least one species of malaria and one species of STH. The majority of coinfections involved two species (2.7%, 12 of 437), followed by three species (2.3%, 10 of 437) and four species (1.1%, 5 of 437). Rare cases involved coinfections with five species (0.2%, 1 of 437) and six species (0.2%, 1 of 437). These findings are further detailed in Supplemental Table 3.

Analysis revealed a significantly higher prevalence of coinfection among individuals with a monthly household income below RM 800 compared with those with higher incomes (*P* = 0.010). Additionally, the Negrito tribe showed a significantly higher coinfection compared with the non-Negrito tribes (*P* <0.001) (Table 3).

## DISCUSSION

This study highlights a 15.3% prevalence of submicroscopic malaria parasitemia cases among the Orang Asli community, despite no reported malaria-related hospitalizations in this population. People residing near forest fringe areas, who are at risk of malaria, may develop partial immunity because of prior exposure, potentially leading to persistent submicroscopic malaria.^39^ Furthermore, recurrent malaria episodes may occur because of recrudescence or relapse, contributing to submicroscopic malaria infections that can reappear months or even years after the initial episode. Limited access to antimalarial drugs often leads to inadequate treatment, which may allow *Plasmodium* parasites to persist at low levels in the body. Any level of malaria parasitemia, regardless of density, can sustain transmission,^40^ posing significant challenge to elimination efforts and adversely affecting the health of infected individuals. Studies show that persistent submicroscopic malaria is associated with ongoing red blood cell destruction^41^ and increased susceptibility to other infections or immune disorders.^42^

Although the country has declared the elimination of indigenous human malaria, *Plasmodium vivax* remained the predominant species in this study, consistent with previous findings.^9^^,^^10^ The persistence of *Plasmodium vivax* and other human malaria parasites (i.e., *Plasmodium falciparum* and *Plasmodium malariae*) continues to challenge malaria elimination efforts. Achieving true malaria elimination requires addressing the full human reservoir of infections, including submicroscopic malaria parasitemia cases. Furthermore, this study found *Plasmodium cynomolgi* as the most common zoonotic malaria species. Previous investigations on submicroscopic malaria parasitemia in Peninsular Malaysia have typically focused on the four human malaria and *Plasmodium knowlesi*, without screening for other simian malaria species,^9^^,^^10^^,^^12^ potentially missing cases of *Plasmodium cynomolgi* and *Plasmodium inui*, both known to infect humans in the country. The high genetic diversity of *Plasmodium cynomolgi* in macaques suggests its potential for adaptation and emergence in local human populations, raising public health concerns.^43^ Accurate estimates of *Plasmodium cynomolgi* cases are challenging because of its morphological similarity to *Plasmodium vivax* and the mild or asymptomatic nature of infections. This study and others emphasize the importance of molecular approaches for identifying *Plasmodium cynomolgi.*^4^^,^^44^^,^^45^

This study also observed submicroscopic malaria parasitemia mixed infections involving up to four *Plasmodium* species in single individuals. Mixed-species infections may arise from *Anopheles* mosquitoes carrying multiple *Plasmodium* species, inoculating them simultaneously or sequentially in one host,^46^ or through reactivation of dormant liver stages of species like *Plasmodium vivax* and *Plasmodium cynomolgi.*^47^ Mixed-species infections, often underreported because of detection challenges,^48^ can affect disease severity, leading to complications like severe anemia, organ damage, and increased mortality.^49^ However, the full impact of submicroscopic mixed-species malaria on morbidity remains unclear.

A higher prevalence of submicroscopic malaria parasitemia was found among females than males in this study. Previous research links higher malaria rates in men to forest-based occupations (e.g., forestry, fishing, agriculture) and exposure during peak mosquito biting hours.^50^^–^^53^ However, women in these communities often engage in similar work, such as on oil palm plantations, putting them at comparable risks. Additionally, women’s domestic activities, like cooking outdoors in the evening or early morning, may increase their exposure.^54^ Individuals aged 50 and older also showed a higher prevalence of submicroscopic malaria parasitemia, possibly because of acquired immunity from past exposure.

On another front, STH infections remain prevalent in the Orang Asli communities, with *Trichuris trichiura* as the predominant species found, consistent with previous research.^16^^,^^17^ A mass deworming program was previously implemented to control and eliminate STH infections among the Orang Asli. Frequent anthelminthic treatments may have contributed to *Trichuris trichiura* drug resistance.^17^ Repeated treatments exert selection pressure, potentially leading to the development of drug-resistant strains and contributing to a higher prevalence of *Trichuris trichiura.*^17^

Economic challenges contribute to poor sanitation, limited healthcare access, and overcrowded living conditions that promote STH transmission.^19^^,^^20^^,^^55^^,^^56^ Dysfunctional toilets because of poor maintenance have led to open defecation practices, amplified by cultural customs involving the use of natural areas, like rivers and bushes, for defecation. This can contaminate water and soil with STH eggs, increasing infection risk. Children are particularly vulnerable because of frequent play in soil and water resources. The combination of environmental exposure and underdeveloped immune systems likely increases susceptibility in this age group.

This study is the first community-based investigation of concurrent malaria and STH infections in Malaysia, revealing an 8.9% coinfection rate of submicroscopic malaria parasitemia and STH among the indigenous population of Peninsular Malaysia, with some cases involving up to six different parasites. Comparable infection rates have been found in other regions, such as 7.1% in Papua, Indonesia,^57^ and 7.0% in southern Ethiopia.^23^ These coinfections can have profound clinical implications, including severe anemia, impaired cognitive function, malnutrition, and increased susceptibility to other infections because of weakened immunity.^23^^,^^58^^–^^61^

Although malaria and STH have distinct transmission pathways, their co-occurrence may be associated with shared risk factors in the Orang Asli communities, such as socioeconomic challenges. Limited financial resources often prevent families from investing in essential mosquito protection measures such as bed nets or window screens. Furthermore, traditional dwellings in these communities offer minimal mosquito protection, and economic limitations lead to inadequate sanitation, poor hygiene infrastructure, and a lack of proper waste management, all conducive to STH transmission.^62^

Another important finding of this study is that the Negrito tribe experiences the highest prevalence of submicroscopic malaria parasitemia, STH infections, and coinfections among the Malaysian Orang Asli. This elevated disease burden correlates with the Negrito’s geographical distribution, as many reside in remote, forested regions on the East Coast and Northern Peninsular Malaysia. These areas, characterized by limited development and urbanization, have lower living standards and often lack essential healthcare infrastructure and basic amenities, compounding health challenges. The Negrito’s isolated lifestyle and traditional forest-dwelling practices further exacerbate these issues.

The government has implemented various control measures through the National Strategic Plan for the Elimination of Malaria to eradicate locally acquired human malaria. Additionally, RM 380 million was allocated in Budget 2025 to improve the Orang Asli’s living standards, including through education.^63^ However, the lack of education and awareness about disease prevention remains a key factor in their vulnerability. Unlike urban populations with better access to health information, many rural communities rely on schools as their primary source of knowledge and information. Unfortunately, school curricula often do not address prevalent infections, such as malaria and STH, and their prevention measures. Integrating health education on malaria, STH, and other endemic infections into school curricula could empower these communities with the knowledge needed to reduce transmission.

## CONCLUSION

The Orang Asli communities in Peninsular Malaysia continue to face considerable health challenges, making it essential to understand the prevalence of parasitic diseases among them for the development of targeted interventions to reduce disease burdens. This study uncovered submicroscopic malaria parasitemia cases detectable only through highly sensitive PCR, underscoring a potential reservoir of transmission that conventional diagnostic methods might overlook. Concurrent STH infections, linked to traditional lifestyle practices and socioeconomic factors, further compound the health burden within the Orang Asli population. If left unaddressed, these infections could significantly affect their overall health and well-being. The findings highlight the need for integrated control strategies targeting both malaria and STH infections, with a focus on the Negrito tribe.

This study has several limitations. Firstly, because of its cross-sectional design, it is challenging to establish a causal relationship between malaria and STH. Longitudinal studies are needed in the future to better understand the interactions between malaria and STH infections and how these parasitic diseases interact within the host. Assessing immune profiles across four groups: those infected only with malaria, only with STH, coinfected with malaria and STH, and uninfected, would provide additional insights.

Furthermore, the lack of infection density data for malaria-positive samples limited the analysis of the relationship between STH infections and malaria severity. Future studies could use quantitative PCR or other sensitive molecular techniques capable of quantifying submicroscopic malaria parasitemia. Another limitation is that although this study examined certain risk factors, it primarily focused on demographic and socioeconomic parameters. It did not explore practice-related factors, such as hygiene, sanitation, personal protection, or vector exposure, that could influence the prevalence of infections. Including these factors in future studies would provide a more comprehensive understanding and help enhance targeted interventions. Additionally, incorporating serological analysis could offer insights into past or recent exposure, immune responses, and factors contributing to susceptibility or resistance to coinfection.

## Supplemental Materials

10.4269/ajtmh.24-0718 Supplemental Materials
