# Supplementary material for: Evidence of Submicroscopic Malaria Parasitemia, Soil-Transmitted Helminths, and Their Coinfections Among Forest-Fringed Orang Asli Communities in Peninsular Malaysia
Source: Am J Trop Med Hyg. 2025 Apr 8;112(6):1391–9. doi: 10.4269/ajtmh.24-0718 (PMC12139561; doi:10.4269/ajtmh.24-0718)
Supplement: Supplemental Materials [file tpmd240718.SD1.pdf]

Supplementary Table S1

Table S1. Distribution of samples across study population.

State	Tribe (Subtribe)	District	Village
Negeri Sembilan	Proto-Malay (Temuan)	Jelebu	F
			G
Melaka		Alor Gajah	E
			D
Selangor		Hulu Langat	C
	Senoi (Mah Meri)	Kuala Langat	A
		Sepang	B
Pahang	Senoi (Jahut)	Temerloh	I
	Negrito (Bateq)	Kuala Lipis	H
Kedah	Negrito (Kensiu)	Baling	J

Supplementary Table S2

Table S2. List of PCR primers sequences and protocols for molecular identification of *Plasmodium* spp.

***Plasmodium* spp.**	**Primer name**	**bp size**	**Annealing temperature (°C)**	**Primer sequence (5' – 3')**
**Genus-specific Nest 1 PCR**				
Genus [31]	rPLU1	1600-1700	55	TCAAAGATTAAGCCATGCAAGTGA
	rPLU5			CCTGTTGTTGCCTTAAACTTC
Nest 2 PCR				
Genus [31]	rPLU3	253	62	TTTTTATAAGGATAACTACGGAAAAGCTGT
	rPLU4			TACCCGTCATAGCCATGTTAGGCCAATACC
**Species-specific Nest 2 PCR**				
P. falciparum [31]	rFAL1	205	58	TTAAACTGGTTTGGGAAAACCAAATATATT
	rFAL2			ACACAATGAACTCAATCATGACTACCCGTC
P. vivax [31]	rVIV1	117	58	CGCTTCTAGCTTAATCCACATAACTGATAC
	rVIV2			ACTTCCAAGCCGAAGCAAAGAAAGTCCTTA
P. malariae [31]	rMAL1	144	58	ATAACATAGTTGTACGTTAAGAATAACCGC
	rMAL2			AAAATTCCCATGCATAAAAAATTATACAAA
P. ovale [31]	rOVA1	787	58	ATCTCTTTTGCTATTTTTTAGTATTGGAGA
	rOVA2			GGAAAAGGACACATTAATTGTATCCTAGTG
P. knowlesi [32]	Pmk8	154	60	GTTAGCGAGAGCCACAAAAAAGCG
	Pmkr9			ACTCAAAGTAACAAAATCTTCC
P. fieldi [33]	PfldF1	421	60	GGTCTTTTTTTTGCTTCGGTAATTA
	PfldR2			AGGCACTGAAGGAAGCAATCTAAGAGTTTC
P. inui [33]	PinF2	479	60	CGTATCGACTTTGTGGCATTTTTCTAC
	INAR3			GCAATCTAAGAGTTTTAACTCCTC
P. cynomolgi [33]	CY2F	137	60	GATTTGCTAAATTGCGGTCG
	CY4R			CGGTATGATAAGCCAGGGAAGT
P. coatneyi [33]	Pctf1	504	60	CGCTTTTAGCTTAAATCCACATAC AGAC
	Pctr1			GAGTCCTAACCCCGAAGGGAAAGG

Supplementary Table S3

Table S3. Prevalence of submicroscopic malaria parasitemia-STH co-infection by species.

**Types of co-infection**	**n**
Overall submicroscopic malaria parasitemia-STH co-infection	39
**Submicroscopic malaria parasitemia-STH polyparasitism (by species)**	
Tt + submicroscopic malaria parasitemia	34
Al + submicroscopic malaria parasitemia	14
Hw + submicroscopic malaria parasitemia	7
**STH + human *Plasmodium***	**10**
Hw + Pv	1
Tt + Pf	1
Tt + Pv	2
Al + Pv	1
Al + Tt + Pv	1
Hw + Tt + Pv	3
Tt + Pf + Pv	1
**STH + zoonotic *Plasmodium***	**8**
Tt + Pk	1
Tt + Pcy	4
Tt + Pi	1
Al + Pk	1
Al + Tt + Pcy	1
**STH + human + zoonotic *Plasmodium***	**11**
Tt + Pv + Pk	1
Tt + Pv + Pcy	3
Tt + Pv + Pk + Pcy	2
Al + Tt + Pv + Pk	1
Ai + Hw + Tt + Pcy	1

Al + Tt + Pv + Pcy	1
Al + Tt + Pv + Pk + Pcy	1
Al + Tt + Pf + Pv + Pk + Pcy	1
**STH + unidentified species of malaria**	**10**
Tt + Ui	5
Al + Ui	1
Al + Hw + Ui	1
Al + Tt + Ui	2
Ai + Hw + Tt + Ui	1
**Total**	**39**

STH: Soil-transmitted helminths; Hw: Hookworm; Tt: *Trichuris trichiura*; Al: *Ascaris lumbricoides*; Pf: *Plasmodium falciparum*; Pv: *Plasmodium vivax*; Pm: *Plasmodium malariae*; Pk: *Plasmodium knowlesi*; Pcy: *Plasmodium cynomolgi*; Pi: *Plasmodium inui;* Ui: Unidentified species
